# The Expression of Thrombospondin-4 Correlates with Disease Severity in Osteoarthritic Knee Cartilage

**DOI:** 10.3390/ijms20020447

**Published:** 2019-01-21

**Authors:** Kathrin Maly, Inna Schaible, Jana Riegger, Rolf E. Brenner, Andrea Meurer, Frank Zaucke

**Affiliations:** 1 Dr. Rolf M. Schwiete Research Unit for Osteoarthritis, Orthopaedic University Hospital Friedrichsheim gGmbH, Marienburgstraße 2, 60528 Frankfurt/Main, Germany; kathrin.maly@friedrichsheim.de (K.M.); Inna.Schaible@friedrichsheim.de (I.S.); andrea.meurer@friedrichsheim.de (A.M.); 2 Division for Biochemistry of Joint and Connective Tissue Diseases, Department of Orthopaedics, University of Ulm, Oberer Eselsberg 45, 89081 Ulm, Germany; jana.riegger@uni-ulm.de (J.R.); rolf.brenner@uni-ulm.de (R.E.B.)

**Keywords:** thrombospondin-4, osteoarthritis, extracellular matrix, biomarker

## Abstract

Osteoarthritis (OA) is a progressive joint disease characterized by a continuous degradation of the cartilage extracellular matrix (ECM). The expression of the extracellular glycoprotein thrombospondin-4 (TSP-4) is known to be increased in injured tissues and involved in matrix remodeling, but its role in articular cartilage and, in particular, in OA remains elusive. In the present study, we analyzed the expression and localization of TSP-4 in healthy and OA knee cartilage by reverse transcription polymerase chain reaction (RT-PCR), immunohistochemistry, and immunoblot. We found that TSP-4 protein expression is increased in OA and that expression levels correlate with OA severity. TSP-4 was not regulated at the transcriptional level but we detected changes in the anchorage of TSP-4 in the altered ECM using sequential protein extraction. We were also able to detect pentameric and fragmented TSP-4 in the serum of both healthy controls and OA patients. Here, the total protein amount was not significantly different but we identified specific degradation products that were more abundant in sera of OA patients. Future studies will reveal if these fragments have the potential to serve as OA-specific biomarkers.

## 1. Introduction

The articular cartilage degeneration in osteoarthritis (OA) is slowly progressing and further hallmarks are the remodeling of the subchondral bone and formation of osteophytes, meaning new bone formation, leading to stiffness and pain [1,2]. The loss of cartilage matrix components in the course of OA alters the molecular composition of the extracellular matrix (ECM) and in that way also its function, meaning its mechanical properties determining the capability of absorbing mechanical forces and enabling a frictionless as well as painless motion [3,4]. Regarding the chronological order of degradation, it is known that proteoglycans (PGs) are affected in an early phase of disease, while collagen breakdown is observed at a much later stage [5]. However, not only the two main suprastructures [6], the collagen network and the aggrecan gel, are degraded but also minor components, like glycoproteins [7,8] and small proteoglycans [9] that bind to and interconnect these structures. In general, proteolytic cartilage matrix degradation releases fragments from the tissue into the synovial fluid that can be detected eventually in the blood circulation. Several proteins and fragments thereof can then be used as biomarkers indicating ongoing cartilage degeneration. However, the appearance of distinct fragments and thus their potential as predictive and/or diagnostic biomarkers depend on the tissue-specific presence of both the substrate as well as the proteolytic enzymes generating unique degradation products.

One protein that is already used as diagnostic marker for OA is the cartilage oligomeric matrix protein (COMP), also referred to as thrombospondin-5. It belongs to the thrombospondin family and binds to several collagen (Col) types as well as to aggrecan and minor components of the cartilage matrix [10,11,12,13,14,15]. As an adaptor protein, it contributes significantly to the stability of the complex network of the ECM [16]. Its concentration in the serum was found to correlate positively with both OA severity and the number of involved joints [17]. However, today we know that COMP serum levels correlate not only with OA but also with other diseases like rheumatoid arthritis [18,19], systemic sclerosis [20], liver disease [21,22], and different forms of cancer [21,23,24], making a specific diagnosis complicated. Therefore, the search for biomarkers fulfilling both being specific for cartilage degeneration in OA and correlating with the severity grade of damage is still ongoing.

A recent analysis of OA patient sera identified specific antibodies against cartilage ECM components [25]. The corresponding antigens might have the potential to serve as biomarkers and, interestingly, antibodies against not only COMP but also against another member of the thrombospondin family, thrombospondin-4 (TSP-4), were detected [25]. TSP-4 and COMP form homo- and heteropentamers and share a similar domain structure [16,26]. TSP-4 is also binding to different types of collagens, including those present in cartilage [10,27].

In contrast to COMP, TSP-4 expression in the joint was mainly described in articular cartilage but not in the growth plate, suggesting a more specific function of TSP-4 in adult articular cartilage [28]. In addition, it was reported recently that thrombospondin-4 expression is increased in bone marrow lesions of OA patients [29]. Further studies indicate that TSP-4 is specifically upregulated during injury and remodeling processes [30,31]. Here, TSP-4 had a protective function in hypertrophy, fibrosis, and inflammation [32], raising the possibility of a similar function in cartilage.

In the current study, we first aimed to systematically characterize the expression and localization of TSP-4 in healthy and OA cartilage. We then analyzed the correlation of TSP-4 expression with OA severity and the potential of TSP-4 to serve as a serum OA biomarker.

## 2. Results

### 2.1. Scoring of Articular Cartilage Samples

**Figure 1:**
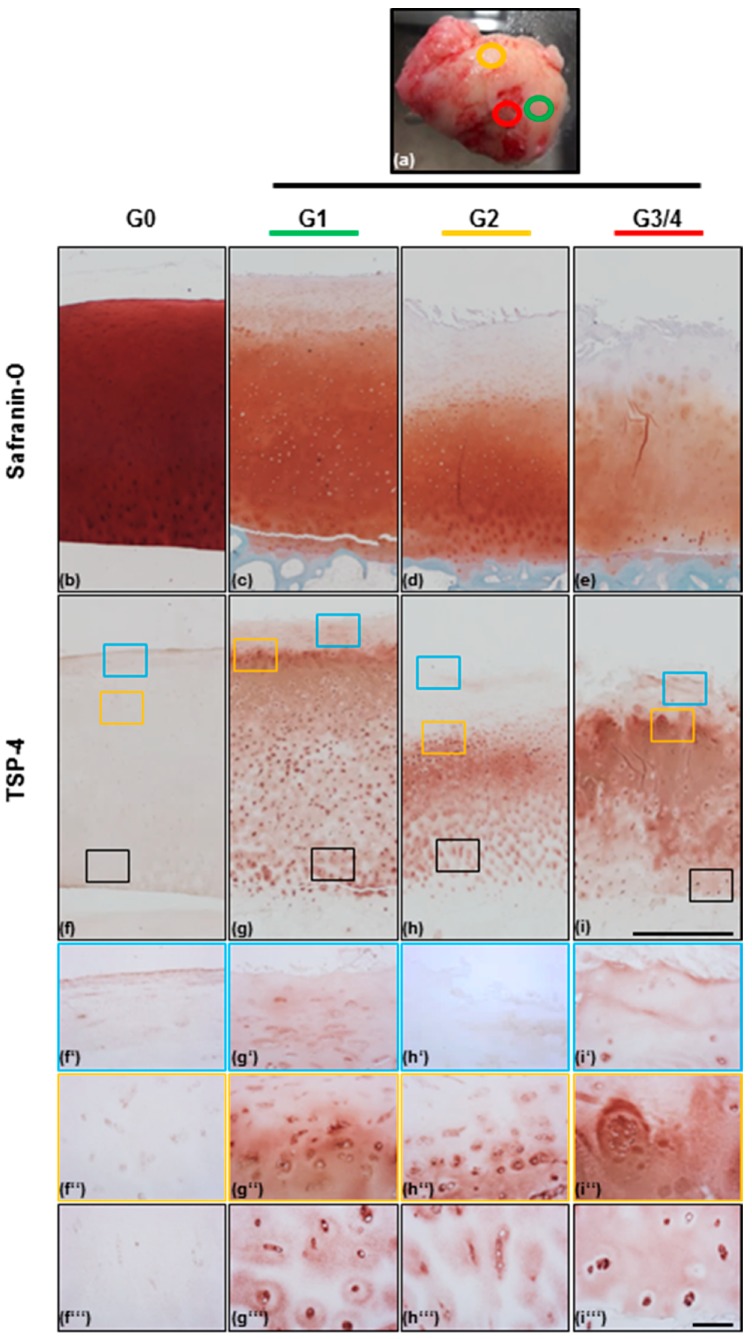
Proteoglycan and thrombospondin-4 localization in non-OA and OA articular cartilage. (**a**) Femoral knee condyle of one OA patient with marked areas, from which osteochondral cylinders were generated after visual grading of the severity grade (green = grade 1 [G1], orange = grade 2 [G2], red = grade 3/4 [G3/4]). Safranin-O/Fast-green staining of proteoglycans in non-OA [G0] (**b**), G1 (**c**), G2 (**d**), and G3/4 (**e**) OA cartilage areas from the knee condyle shown above (**a**). Proteoglycan degradation correlates with OA severity. TSP-4 levels are increasing from G0 (**f**) to G1 (**g**), G2 (**h**), and G3/4 (**i**) OA cartilage. 40× magnification of the boxes (blue = superficial zone, orange = transition zone, black = deep zone) drawn in picture (**f**–**i**), showing the differential distribution of TSP-4 in surface areas (**f’**–**i’**), transition zone of most intensive staining (**f’’**–**i’’**), and deep areas (**f’’’**–**i’’’**) of OA cartilage. Pictures were shown as representative data from different donors. Scale bar (**b**–**i**): 1mm; (**f’**–**i’’’**): 100 µm.

In total, we obtained 21 articular cartilage samples from patients undergoing endoprosthetic knee replacement surgery at the Orthopaedic University Hospital Friedrichsheim, Frankfurt/Main, Germany. The morphological appearance of the cartilage was graded visually based on the scoring system of the Osteoarthritis Research Society International (OARSI) (Figure 1a). The surface of the knee condyle was observed and intact cartilage with a smooth surface and no fissures was scored as grade 1 (G1). Cartilage with superficial discontinuities and fissures was scored as grade 2 (G2), and if deep fissures were present or the subchondral bone was already exposed, cartilage was categorized as grade 3 and grade 4, respectively (G3 or G4). The amount of cartilage received from areas of G3 and specifically G4, was rather limited and, therefore, grade 3 and grade 4 were combined to G3/4.

### 2.2. TSP-4 Expression and Localization in Human Articular Cartilage 

Histological staining of articular cartilage with Safranin-O was performed to demonstrate the presence and localization of proteoglycans (PGs). According to the literature, PGs were intensely and homogenously stained in non-OA (grade 0; G0) cartilage and started to be continuously degraded from G1 to G3/4 OA cartilage (Figure 1b–e). This overview staining of PGs was also used to confirm the first classification of the samples that was done by visually grading the morphological appearance of femoral condyles. In G3/4 cartilage the superficial and upper zone is already degraded and cell clusters were seen (Figure 1i’’). For the detection of TSP-4, we used a specific antibody raised in rabbits and directed against the recombinant full-length protein. TSP-4 was almost undetectable in healthy cartilage, with only a very weak staining in the superficial zone (blue boxes). All OA tissues were obviously more intensely stained than the healthy cartilage. In all severity grades, the most intense TSP-4 staining was seen in the transition zone (yellow boxes) in which PGs were still present but degradation was already initiated. The stained area expands with severity grade and the borders become blurred. TSP-4 was present in all areas of the ECM in the transition zone and it seems as if there was an intracellular staining. Compared to the transition zone, the interterritorial staining became weaker in deeper cartilage layers (black boxes). Further, a stronger pericellular and intracellular localization was seen. The overall staining intensity seems to increase with severity (Figure 1f–i). In addition, minor differences in the protein localization depending on severity could be detected (Figure 1f’–i’’’). In the deep zone, there is an increased signal in the interterritorial matrix in G3/4 as compared to G1 and G2 (Figure 1f’’’–i’’’).

### 2.3. Total TSP-4 Protein Amount Increases with OA Severity Grade

**Figure 2:**
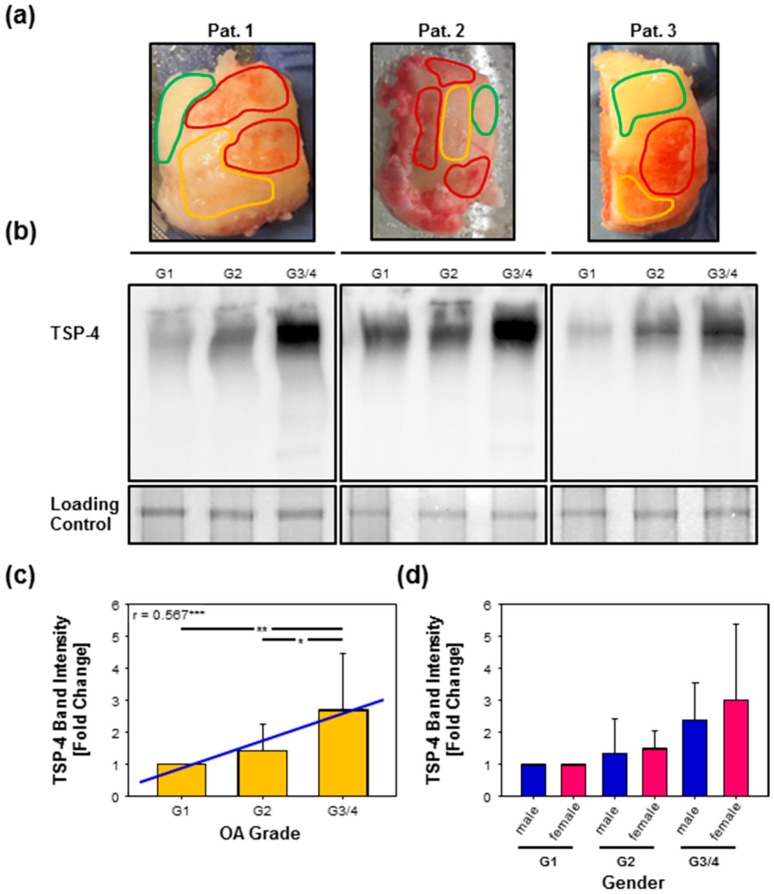
Detection of TSP-4 in total protein extracts from OA knee articular cartilage. (**a**) Knee condyles from three OA patients with marked areas, from which the proteins were isolated (green = grade 1 [G1], orange = grade 2 [G2], red = grade 3/4 [G3/4]). (**b**) Proteins extracted from areas of different OA severity grades were analyzed via immunoblot to detect TSP-4. Equal loading was demonstrated via PageBlue^TM^ staining (loading control). (**c**) Statistical analysis of the immunoblots revealed an increase of TSP-4 with OA severity grade. The amount of TSP-4 correlated positively with OA severity (r = 0.567, ***, blue line). (**d**) No difference in TSP-4 levels between male and female OA patients was found at any severity grade. Immunoblots were shown as representative data from different donors. Values are represented as means ± SD and significance (*p* < 0.05 *; *p* ≤ 0.01 **; *p* < 0.001 ***) was analyzed by Friedman test with Tukey post hoc analysis or Mann–Whitney U test as well as the correlation with the Spearman rank test. Pat. = patient; OA = osteoarthritis.

In order to evaluate total TSP-4 protein amounts in cartilage quantitatively, we performed protein extractions followed by immunoblots. A specific antibody raised against recombinantly expressed full-length TSP-4 [27,33] was used to detect TSP-4. The pentameric protein has a theoretical molecular mass of ~700 kDa. In order to analyze TSP-4 expression in different stages of OA, total proteins were extracted from the tissue samples of ten patients (Figure 2a). The detection of total TSP-4 showed a certain variability between individual patients, but was always increasing from G1 to G3/4 (Figure 2b). To minimize this variability, we calculated the fold change of G2 and G3/4 to G1. The analysis revealed an increase in TSP-4 level from G1 to G3/4 (*p* = 0.01 **) and from G2 to G3/4 (*p* = 0.037 *) but not from G1 to G2 (*p* = 0.869). Furthermore, the increase of TSP-4 protein level correlated positively with OA severity grade (*p* < 0.001 ***; r = 0.567) (Figure 2c). No difference in the level of TSP-4 could be observed, at any severity grade, between male and female patients (Figure 2d).

### 2.4. Analysis of TSP-4 Anchorage in OA Cartilage

**Figure 3:**
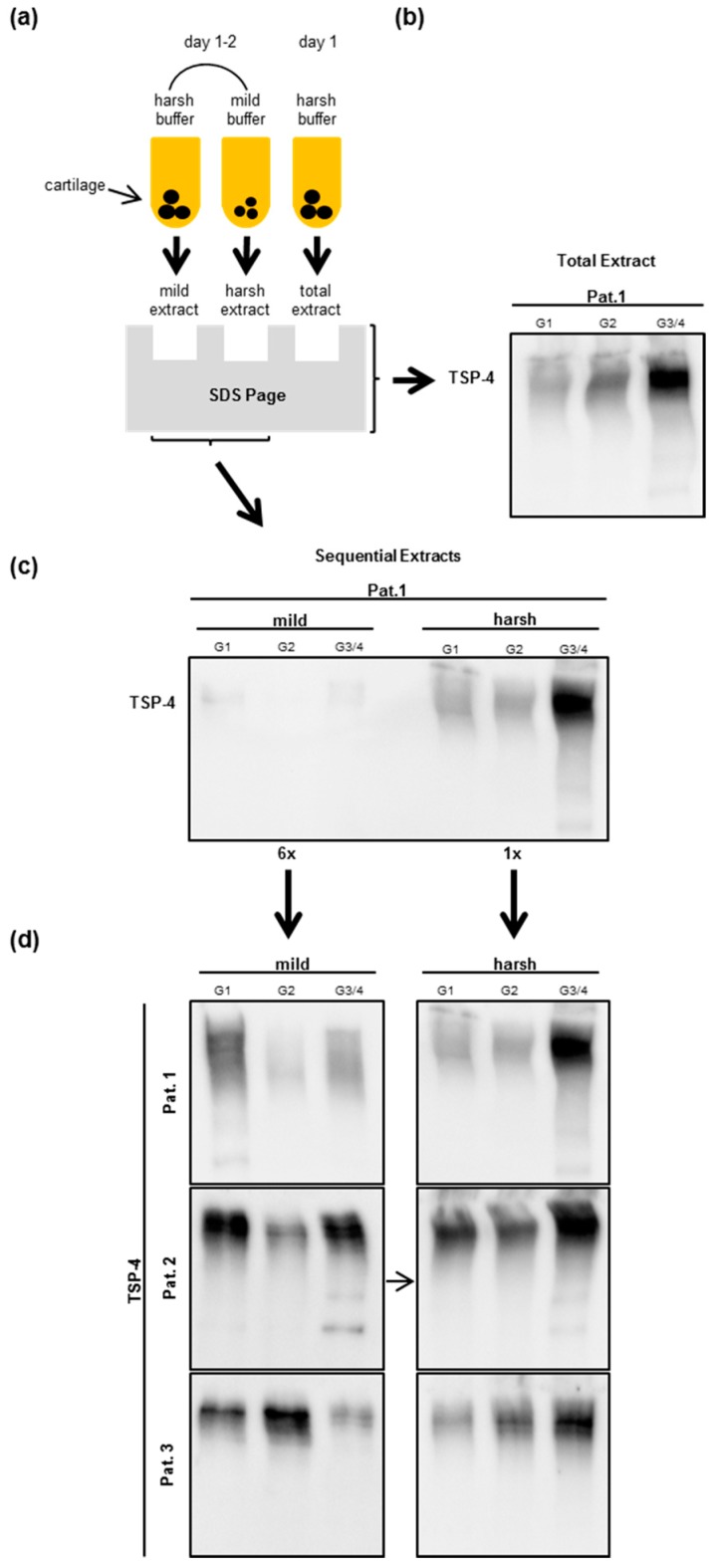
Detection of TSP-4 in extracts after sequential protein extraction from OA knee articular cartilage. (**a**) Schematic overview of the sequential protein extraction procedure. On the first day, proteins were extracted overnight with a mild buffer and supernatants were collected on the following day. Remaining cartilage pieces were resuspended in a harsh buffer to extract still-anchored proteins. To extract total proteins, only the harsh buffer was added and the total protein extract collected. Total (**b**), weakly, and tightly anchored TSP-4 (**c**) level in OA patients were analyzed via immunoblot. When equal protein amounts were loaded, the weakly anchored proteins were hardly detectable. Therefore, a six-fold amount of this extract was loaded to be able to detect differences between OA severity grades and to compare the extraction behavior with that of tightly anchored proteins (**d**). Immunoblots were shown as representative data from different donors. OA severity grades: grade 1 (G1), grade 2 (G2), and grade 3/4 (G3/4). Pat. = patient.

To analyze the anchorage of TSP-4 in the ECM depending on the OA severity grade, we extracted proteins sequentially from OA cartilage. First, we used a mild buffer to extract soluble and weakly anchored proteins. This was followed by a second extraction of the same piece of cartilage tissue with a harsh buffer to extract all remaining and tightly anchored proteins. In this second extraction step, we used the same buffer as for the total protein extraction (Figure 3a,b). When loading the same amount of total protein, we could not see a clear signal after the first mild extraction while specific bands could be detected after extracting under harsh conditions (Figure 3c). Therefore, we had to load six times the amount of proteins extracted under mild conditions to allow a comparison of bands between the severity grades. Obviously, only a minimal proportion of total TSP-4 is weakly anchored. The profile of the second extraction was very similar to the profile of a single-step total TSP-4 extraction (Figure 3b,d). We were not able to detect a clear and consistent difference between the severity grades in the amount of proteins extractable under mild conditions (Figure 3d). The amount of TSP-4 which was extracted with the harsh buffer increased from G1 to G3/4 and from G2 to G3/4 in all patients (Figure 3d). In summary, this means that the extractability of tightly anchored TSP-4 depends on the severity grade, while this does not apply for less well anchored TSP-4. No differences in protein anchorage could be seen between male and female, at any stage of OA (data not shown).

### 2.5. Gene Expression of Thbs-4 Is Not Increasing During OA

**Figure 4:**
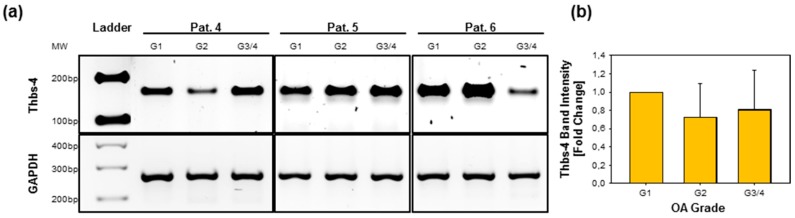
Thrombospondin-4 gene expression in OA knee articular cartilage. (**a**) *Thbs-4* gene expression was analyzed by RT-PCR, followed by agarose gel electrophoresis. The *Thbs-4* amplicons were normalized to the housekeeper GAPDH. Representative gels of RNA isolated from three different donors are shown. (**b**) No difference in gene expression could be detected when comparing the band intensities for different OA severity grades normalized to G1 (=1). Values are presented as means ± SD and significance (*p* < 0.05 *; *p* < 0.01 **; *p* < 0.001 ***) was analyzed by Friedman test with Tukey post hoc analysis. OA severity grades: grade 1 (G1), grade 2 (G2), and grade 3/4 (G3/4). Pat. = patient; OA = osteoarthritis.

To investigate if the increased TSP-4 protein level in severe OA is caused by an increased transcriptional activity, we converted RNA isolated freshly from cartilage of different severity grades into cDNA. Using specific primers for thrombospondin-4 (Thbs-4) as well as for the housekeeping gene glycerinaldehyd-3-phosphat-dehydrogenase (GAPDH), we were able to amplify bands of the expected size (Figure 4a). No significant differences between severity grades with regard to the level of Thbs-4 gene expression could be deduced from band intensities. G2 or G3/4 samples from some patients showed a slight decrease in signal intensity of the corresponding bands but none of the tested patients showed an increase in late-stage OA (Figure 4b).

### 2.6. TSP-4 Levels Were Increased in Serum of OA Patients

**Figure 5:**
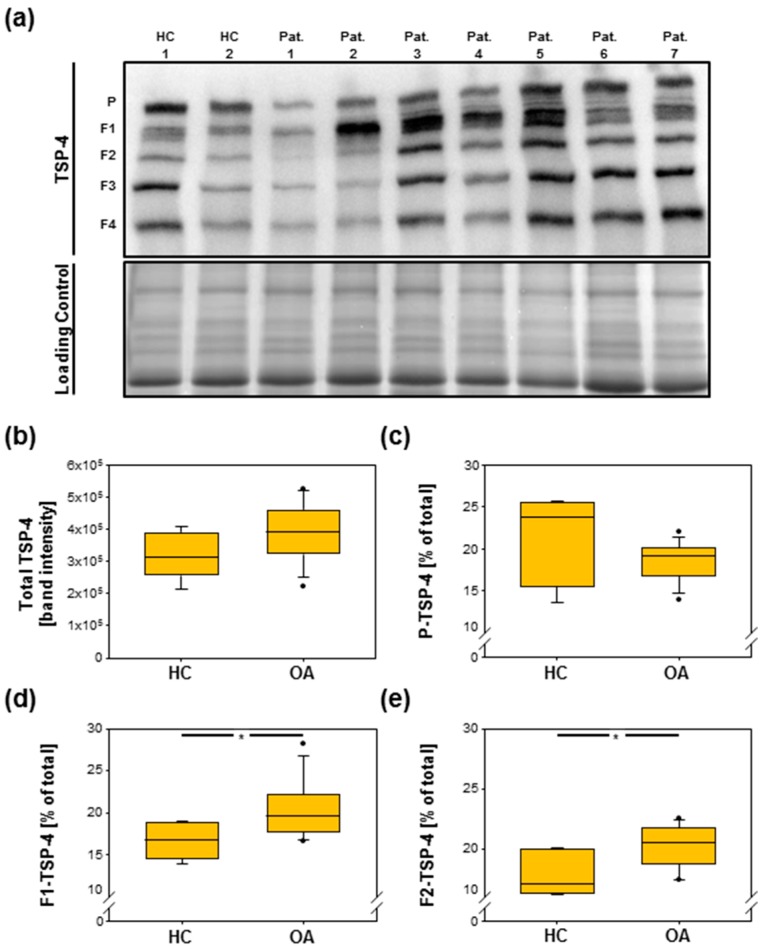
Detection of TSP-4 in sera of healthy donors and OA patients. (**a**) The immunoblot analysis of sera shows the pentameric TSP-4 and derived fragments (1–4). Equal loading was demonstrated via PageBlue^TM^ staining (loading control). Amounts of total (**b**) as well as of pentameric TSP-4 (**c**) are not significantly different between HCs and OA, but a significant increase of fragment 1 (**d**) and fragment 2 (**e**) could be detected in OA patients. Immunoblot was shown as representative blot. Values are represented as box blots and significance (*p* < 0.05 *) was analyzed by the Mann–Whitney U test. Dots in figures b-e represent outliers. HC = healthy controls; Pat. = patient; OA = osteoarthritis; P = pentamer, F = fragment.

To analyze TSP-4 levels in serum of healthy controls (HCs) and OA patients, we performed immunoblots. We found that pentameric TSP-4 as well as fragments of TSP-4 were present in the sera of both HCs and OA patients (Figure 5a). We did not detect significant differences in the amount of total (*p* = 0.151) (Figure 5b) and pentameric (*p* = 0.375) (Figure 5c) TSP-4, even though there might be a tendency for the amount of the pentamer to be slightly increased in HCs compared to patients. However, amounts of fragment 1 (*p* = 0.03 *) (Figure 5d) as well as of fragment 2 (*p* = 0.023 *) (Figure 5e) were significantly increased in OA patients. The levels of fragment 3 (*p* = 0.844) and fragment 4 (*p* = 0.139) were not altered in OA.

## 3. Discussion

The role of thrombospondin-4 in OA is largely unknown and there are no data available about its localization in human articular cartilage, neither in healthy donors nor in OA patients. Jeschke et al. [28] showed in a mouse model that TSP-4 is only expressed in articular cartilage but not in the growth plate, suggesting a unique role in adult but not in developing cartilage. Especially, during tissue injury or under pathological conditions, like OA, the expression of certain proteins is often found to be increased and/or reinduced. This has been shown in previous studies for several matrix proteins such as type II collagen [34], COMP [35], and matrilin-3 [7], and is now also reported in the present study for TSP-4. 

TSP-4 is only very weakly expressed in healthy cartilage and was hardly detectable on tissue sections. Its localization is restricted to the superficial zone, which is characterized by its high tensile strength and stiffness [36,37] to protect the subjacent layers [38]. The superficial layer is exposed to shear stress and mechanical forces [38] and it can be assumed that the mechanical properties of this zone will change with progression of OA. The expression of several cartilage matrix proteins, including the abovementioned and TSP-4-related protein COMP, has been shown to be mechanosensitive [39]. Even if the molecular function of TSP-4 is largely unknown, it is attractive to speculate that it also contributes to the mechanical stabilization of the cartilage ECM. In particular, it has been shown to respond to substrate stiffness and mechanical alterations in tendon and muscle tissue [40,41].

Compared to the low abundance in healthy articular cartilage, TSP-4 levels are strongly upregulated in OA tissue and, interestingly, we found a positive correlation of TSP-4 levels with OA severity implying a disease-associated role. In tissue repair and regeneration, rapid upregulation of TSP-4 was described. The authors speculated that TSP-4 might be involved in the regulation of matrix production and remodeling [31,42,43], both processes that are also highly relevant in OA pathogenesis. 

In OA, TSP-4 localization is not restricted to a specific zone in cartilage, but is predominantly found in the interterritorial matrix of the transition zone where PGs become degraded first. So far, it has not been studied if TSP-4 interacts directly with proteoglycans, like aggrecan. However, as other thrombospondin family members do so, it is highly likely that TSP-4 shares not only a similar structure but also this function [15,44]. The reinduction of other matrix proteins has been interpreted primarily as an attempt to prevent or at least to decelerate tissue degeneration [34,45,46]. TSP-4 might also compensate for the loss of other matrix components, including PGs, and exert a protective effect by contributing to the stabilization of the matrix network. 

Besides its interterritorial localization, TSP-4 was detected in the pericellular matrix, which is important for cellular signaling but also for sequestering of proteins like growth factors and enzymes [47]. A major component of the pericellular matrix is Col VI, which is also enriched in OA [47,48,49,50]. Together with Col VI, TSP-4 might stabilize the pericellular matrix and protect the chondrocytes from interacting with small molecules, like degradation products [50], and against mechanical loading [51]. It remains to be determined if TSP-4 and Col VI can interact directly, but it is already known that a variety of other collagen types are binding partners [27]. In OA, nidogen-2 and laminins were also found to be increased in the pericellular matrix. Laminins were shown to induce Col II, COMP, and aggrecan expression [45]. As TSP-4 interacts with laminin-1 [27], it could also play a regulatory role here. 

Even though we found increased total TSP-4 protein amounts in cartilage tissue correlating with OA severity, we did not detect alterations in TSP-4 expression at the RNA level. This could be explained by an extended half-life or slower turnover of the protein. Another possibility is an altered anchorage of the protein in the ECM. Due to the degradation of other components, additional binding sites for TSP-4 might become available. A similar phenomenon was reported in the ECM of several genetically modified mouse lines where the absence of a single distinct matrix component altered the anchorage of other proteins [52,53,54]. To address this issue experimentally, we analyzed the anchorage of TSP-4 in OA cartilage by extracting proteins using mild and harsh conditions. Provided that the TSP-4 anchorage is unchanged, one would expect a similar extraction profile with each extraction buffer used and the same protein ratio between mild and harsh extraction. However, we found that the amount of extractable protein under harsh conditions was always highest in G3/4 while this was not the case under mild conditions.

This indicates that TPS-4 is most tightly anchored in G3/4 cartilage and that the anchorage depends on severity grade. Based on this, we conclude that TSP-4 is preferably degraded or released in an early stage of OA, while it is more tightly integrated and accumulated in the ECM at later stages. We speculate that a different anchorage is caused by changes in the number of available binding sites for TSP-4 but cannot predict how other proteins with different binding sites would behave. 

Even though we did not detect changes in RNA expression, we observed an intracellular staining of TSP-4 in cartilage tissues of all three OA severity grades, pointing to an intracellular function in addition to its structural role. Intracellularly, TSP-4 was shown to be involved in the endoplasmic reticulum (ER) stress response [55], acting as a chaperone in protein folding and trafficking as well as secretion [55,56]. More related to the ECM, it was shown that TSP-1 is involved in Col I processing and assembly [57]. A similar role that has been reported earlier for COMP is involvement in the export of collagen molecules from the ER and the following assembly into fibers within the extracellular matrix. As a consequence of this intracellular function, COMP-deficient mice showed retention of procollagens [58]. Recently, it was demonstrated that COMP and TSP-4 bind fibrillar collagens via the same binding site [10], further strengthening the hypothesis that TSP-4 and COMP might have a similar function in collagen trafficking. Increased levels of TSP-4 could be important for an increased expression and/or secretion of fibrillar collagens like Col II [34] but maybe also of nonfibrillar Col VI [49] and Col X [59], which are all known to be upregulated in OA. 

OA is characterized by an increased activity of matrix-degrading enzymes like matrix metalloproteinases (MMPs) and/or ADAMTS (a disintegrin and metalloproteinase with thrombospondin motifs). Due to their activities, various degradation products are generated and released from the tissue. These degradation products eventually reach the serum and might serve as markers for cartilage degeneration. As we found increased levels of TSP-4 protein in the tissue and an altered anchorage of the protein in different stages of OA, we analyzed TSP-4 in serum of OA patients using immunoblots. The level of total and pentameric TSP-4 in the serum was comparable in HC and OA patients. This is in accordance with an earlier study [28], where no significant differences between HC and patients with either mono- or poly-OA were detected using an enzyme-linked immunosorbent assay (ELISA). It might be that significance could have been reached by increasing the numbers of samples. In the present study, we focused on specific fragments that are most likely generated by specific OA-relevant proteases. It has been shown earlier for other matrix proteins that specific neoepitopes can add valuable information [60,61]. In our study, two specific TSP-4 fragments (fragment 1 and 2) were found increased in OA compared to HC sera, suggesting a specific degradation pattern in OA cartilage. The amount of fragments 3 and 4 were not changed, indicating an OA-independent cleavage of TSP-4. The fragments have to be characterized biochemically and functionally in more detail and future studies will show if these fragments can induce the expression of both inflammatory mediators as well as matrix-degrading enzymes, thereby perpetuating the cartilage degeneration as shown for other matrix proteins [62,63]. To our knowledge, there are no data available about the mechanisms of TSP-4 degradation. Due to its structural similarities to other members of the thrombospondin family, we are hypothesizing that TSP-4 could be cleaved by MMP-9, MMP-13, ADAMTS-7, and ADAMTS-12, which are all increased in OA [64,65,66] and able to cleave COMP [67,68,69]. However, further studies will show which enzymes indeed cleave TSP-4 and what effects the resulting fragments have on chondrocytes and also cartilage.

In summary, we report that TSP-4 is present in articular cartilage and increases dramatically in OA, where it might contribute to the maintenance of cartilage structure and function. TSP-4 levels in cartilage correlate positively with OA severity and the anchorage of TSP-4 in the matrix seems to be weaker at an early disease stage. TSP-4 can be detected in serum and even though the total amount in OA remains unchanged, we found two specific TSP-4 fragments that are increased in patient sera. Future studies will show if these fragments have the potential to serve as biomarkers for OA.

## 4. Materials and Methods

### 4.1. Collection of Clinical Species

Adult, human, anonymized cartilage samples were obtained from 21 patients undergoing endoprosthetic knee replacement surgery at the Orthopaedic University Hospital Friedrichsheim, Frankfurt/Main, Germany. The mean age of patients was 69 years (45–87 years) and included 6 males and 15 females. Two non-OA control cartilage samples for (immuno-)histological staining were a gift from Gerjo van Osch (Erasmus MC University Rotterdam, Rotterdam, Netherlands) and one cartilage sample was obtained from a trauma patient treated at the Orthopaedic University Hospital Friedrichsheim (Frankfurt/Main, Germany). The mean age of control samples was 39 years (min. = 17 years; max. = 52 years). 

Serum samples were used from a previous study [70]. In total, 18 serum samples from either HCs (n: 5; age: 39–64 years; gender: 4 males and 1 female) and knee OA patients (n: 13; age: 54–65 years; gender: 8 males and 5 females) were used for analysis.

### 4.2. Grading of Cartilage Samples

First, all femoral cartilage condyles were visually scored according to the following criteria, based on the OARSI grading system [71]. Cartilage areas with a smooth surface were scored as grade 1 (G1), cartilage areas with superficial fissures were scored as grade 2, and areas with deeper fissures and/or exposure of subchondral bone as grade 3 to 4 (G3/4). To confirm the severity grade histologically, we stained paraffin-embedded cartilage sections with Safranin-O and Fast-green (for details see below “Histological staining”). Non-OA controls were categorized as grade 0 (G0).

### 4.3. (Immuno-)Histological Analysis of Cartilage Samples

Osteochondral cylinders of 7 OA patients (n: G1 = 7, G2 = 5, G3/4 = 5; age: 50–85 years; gender: 2 male and 5 female) were generated, fixed in 4% paraformaldehyde in phosphate-buffered saline (PBS), pH = 7.4, overnight at 4 °C. Samples were decalcified in 10% ethylenediaminetetraacetic acid (EDTA) and embedded in paraffin. Sections of 5 µm were deparaffinized and rehydrated prior to staining of proteoglycans with Safranin-O and Fast-green. Slides were first stained for 5 min with Weigert’s Iron Hematoxylin and washed 4 times in dH_2_O, before treating with 1% acetic alcohol for 2 s. After another 3 rinses in dH_2_O, slides were incubated in 0.02% Fast-green for 1 min, 1% acetic acid for 30 s, and 1% Safranin-O for 30 min. After a brief rinse in 95% ethanol (EtOH), tissues were dehydrated (3× 95% EtOH, 2× 100% EtOH, and 3× xylene) and coverslipped with a glycerole–gelatine mounting media. 

For immunohistological staining, tissues were deparaffinized and rehydrated before antigen retrieval with hyaluronidase (250U; Sigma Aldrich, St. Louis, MO, USA, # H3506,) in PBS (pH = 5) for 15 min at 37 °C. Prior to primary antibody incubation, the endogenous peroxidase activity was blocked with 0.3% H_2_O_2_ (Carl Roth, Karlsruhe, Germany) in dH_2_O for 10 min at room temperature (RT) and blocked with 2.5% normal horse serum (included in the ImmPRESS^TM^ HRP Reagent Kit, Vector Laboratories, Burlingame, CA, USA) for 20 min at RT. After incubation with a rabbit anti-rat antibody raised against recombinantly expressed full-length TSP-4, diluted 1:500 in 1% bovine serum albumin (BSA) overnight at 4 °C, the tissues were incubated with ImmPRESS™ (peroxidase) polymer anti-rabbit IgG reagent (included in the ImmPRESS^TM^ HRP Reagent Kit, Vector Laboratories, Burlingame, CA, USA) at RT for 30 min. For detection, the AEC-2-component kit (DCS, Hamburg, Germany) was used according to the manufacturer’s instructions with 3-amino-9 ethylcarbazole as chromogen. Negative control stainings without addition of the primary antibody were carried out to exclude unspecific binding of the secondary antibody (data not shown).

### 4.4. Protein Extraction from Articular Cartilage

Articular cartilage of knee condyles was scraped off from areas with specific OA severity grades (G1, G2, and G3/4). Only patients showing all three severity grades were included (n: 10 in each group; age: 47–87 years; gender: 5 male and 5 female). The cartilage from areas with different severity grades was separately washed with PBS and cut into pieces (1–2 mm^3^). Cartilage pieces were transferred into tubes, weighed, and either processed immediately or stored until further usage at −80 °C. Proteins were sequentially extracted, first with a mild buffer (0.15 M NaCl, 50 mM Tris [pH = 7.4]) followed by a harsh buffer (4 M Guanidine Hydrochloride, 50 mM Tris, 10 mM EDTA [pH = 7.4]) overnight at 4 °C on a rotator at 40 rpm. 10 volumes (mL per gram of wet tissue) of chilled buffer were added. All buffers contained 2 mM phenylmethylsulfonyl fluoride and 2 mM N-ethylmaleimide. After 24 h incubation with the mild buffer, the extracts were clarified by centrifugation and stored at −20 °C. The cartilage pieces were re-extracted in an identical manner with the harsh buffer. We also extracted the total protein amount by adding only the harsh buffer [54].

Proteins extracted with the mild and/or the harsh buffer (sequential and total) were precipitated with 10 times the volume of 96% EtOH for 24 h at −20 °C. The proteins were separated from the supernatant by centrifugation and the pellet washed with a mixture of 9 volumes of 96% EtOH and 1 volume tris-buffered saline (TBS) for 2 h at 4 °C with gentle agitation. After centrifugation, the pellets were air dried and resuspended in 1× Laemmli buffer (4× Laemmli buffer: 8% sodium dodecyl sulfate (SDS), 40% glycerol, 0.04% bromophenol blue, 250 mM Tris-HCl pH = 6.8). Protein aliquots were cooked for 5 min at 95 °C before loading and stored at −20 °C.

### 4.5. SDS-Polyacrylamide Gel Electrophoresis (PAGE) and Immunoblot Analysis 

Protein extracts from OA cartilage and serum samples (5 µL) of OA patients and healthy controls were separated by SDS-PAGE using 5% or 8% polyacrylamide gels. The separation was carried out using a Mini-PROTEAN^®^ Tetra-cell system (Bio-Rad, Munich, Germany) at 200 V. Equal protein loading was demonstrated on 8% polyacrylamide gels that were washed 3 × 10 min with ddH_2_O and incubated with a PageBlue™ Protein Staining Solution (ThermoFisher Scientific, Waltham, MA, USA) overnight at RT with gentle agitation. After electrophoresis, proteins were transferred onto a 0.45µm polyvinylidene fluoride (PVDF) membrane (GE Healthcare, Freiburg, Germany) using the mini Trans-blot^®^ electrophoretic transfer cell (Bio-Rad, Munich, Germany). After blocking with 10% skim milk for 1 h at RT with gentle agitation, the membranes were incubated with a polyclonal serum of a guinea pig immunized with recombinantly expressed full-length TSP-4 (rat) [27,33] overnight at 4 °C. We used the same antibody to detect TSP-4 in serum and cartilage samples. Polyclonal rabbit anti-guinea pig immunoglobulins/HRP antibody (Agilent Technologies, Little Falls, CA, USA, # P014102-2) was used as secondary antibody and the membranes incubated for 1 h at RT. Blots were detected by using a mixture of a homemade ECL solution (0.1 M Tris-HCl, pH = 8.5; 225 mM p-coumaric acid; 1.25 mM luminol) with 3% H_2_O_2_. The membranes were then analyzed with the Chemi Doc^TM^ XRS+ (Bio-Rad, Munich, Germany) molecular imager and the ImageLab^TM^ software (http://www.bio-rad.com/de-de/product/image-lab-software) and the band intensities quantified with the ImageJ version 1.5 software (http://imagej.nih.gov/ij). To demonstrate the specificity of the primary antibody, tendon extracts from wild-type and TSP-4 knockout mice were used (data not shown).

### 4.6. RNA Isolation and cDNA Synthesis

RNA was isolated from chondrocytes to obtain a higher yield compared to direct isolation from cartilage tissue with the NucleoSpin^®^RNA/Protein purification kit according to the manufacturer’s manual (Macherey-Nagel, Düren, Germany). Articular cartilage of knee condyles was scraped off from areas with specific OA severity grades (G1, G2, and G3/4). Only patients showing all three severity grades were included (n: 6 in each group; age: 57–77 years; gender: 0 male and 6 female). The cartilage of each severity grade was separately washed with PBS and cut into pieces (2–3 mm^3^) to isolate chondrocytes. The cartilage pieces were weighted, transferred into a sterile tube, and digested with 0.2% (weight per volume [*w*/*v*], g/mL) pronase (Roche Diagnostics, Mannheim, Germany) in Dulbecco’s Modified Eagle’s Medium (DMEM)/F12 medium (complete medium contains 5% penicillin/streptomycin and 5% fetal bovine serum) for 2 h at 37 °C with an agitation of 60 rpm. After incubation, cells and cartilage pieces were pelleted by centrifugation (300× *g*, 5 min; Mega Star 3.0, VWR, Osterode am Harz, Germany) and the supernatant decanted. Cells and cartilage pieces were washed 3× with PBS and digested with collagenase type II (200 U/mL, Biochrom, Berlin, Germany) solution in complete DMEM/F12 medium overnight at 37 °C with an agitation of 60 rpm. The chondrocyte suspension was filtered through a 70 µM nylon cell strainer (Corning, New York, NY, USA) and centrifuged. The pelleted cells were washed twice with PBS and finally centrifuged at 17,000× *g* for 5 min at 4 °C (Micro Star 17R, VWR, Osterode am Harz, Germany) as well as stored at −80 °C.

### 4.7. Polymerase Chain Reaction and Agrose Gel Electrophoresis

RNA was converted into cDNA with reverse transcriptase by using the qScript cDNA supermix (Quanta BioSciences, Beverly, MA, USA) according to the manufacturer’s manual in the qTOWER^3^G real-time PCR thermal cycler (Analytik Jena AG, Jena, Germany). PCR was performed in a 20 µL reaction, containing 2 µM each of primer (forward and reverse), cDNA (6–10 ng RNA), and Taq PCR master mix (Qiagen, Hilden, Germany). For all primers, the same PCR program was used. Step 1: 94 °C for 1 s and 36 cycles of each of the following steps. Step 2: 94 °C for 30 s, step 3: 64 °C for 30 s, and step 4: 72 °C for 60 s. GAPDH (forward: CTCCTGTTCGACAGTCAGCC, reverse: TTCCCGTTCTCAGCCTTGAC; product length = 262 bp) and thrombospondin-4 (forward: ATGAAGGCTCTGAGTTGGTG, reverse: CTTGGAAGTCCTCAGGGATG; product length = 153 bp) primers (ThermoFisher Scientific, Waltham, MA, USA) were used. PCR amplicons were analyzed on a 1.8% (*w*/*v*) agarose gel containing GelRed nucleic acid gel stain (Biotium Inc, Hayward, CA, USA). The gels were analyzed with the Chemi Doc^TM^ XRS+ (Bio-Rad, Munich, Germany) molecular imager and the ImageLab^TM^ software (http://www.bio-rad.com/de-de/product/image-lab-software) and the band intensities quantified with the ImageJ version 1.5 software (http://imagej.nih.gov/ij). The band size of every PCR amplicon was counterchecked with the expected band size to validate specificity.

### 4.8. Statistical Analysis

Statistical analysis was performed using SigmaPlot version 13.0 software (Systat Software, Inc, San Jose, CA, USA; https://systatsoftware.com/downloads/). Differences between groups were evaluated by Friedman test or Mann–Whitney U test with the Tukey post hoc test. Correlations between groups were analyzed by using the Spearman rank test (r). A *p*-value < 0.05 was considered as significant difference (*p* < 0.05 *; *p* ≤ 0.01 **; *p* ≤ 0.001 ***).

## Abbreviations

ADAMTS	A disintegrin and metalloproteinase with thrombospondin motifs
BSA	Bovine serum albumin
cDNA	Complementary DNA
Col	Collagen
COMP	Cartilage oligomeric matrix protein
DMEM	Dulbecco’s Modified Eagle’s Medium
ECM	Extracellular matrix
EDTA	Ethylenediaminetetraacetic acid
ELISA	Enzyme-linked immunosorbent assay
EtOH	EtOH
ER	Endoplasmic reticulum
F	Fragment
Fig	Figure
G	Grade
GAPDH	Glycerinaldehyd-3-phosphat-dehydrogenase
HC	Healthy controls
HRP	Horseradish peroxidase
MMP	Matrix metalloproteinase
OA	Osteoarthritis
P	Pentamer
Pat	Patient
PBS	Phosphate-buffered saline
PCR	Polymerase chain reaction
PG	Proteoglycan
SDS	Sodium dodecyl sulfate
SDS-PAGE	Sodium dodecyl sulfate polyacrylamide gel electrophoresis
RT	Room temperature
RT-PCR	Reverse transcription polymerase chain reaction
TGF-β	Transforming growth factor-β
TBS	Tris-buffered saline
TSP-4	Thrombospondin-4
*w*/*v*	Weight per volume
